# Diversity and Plant Growth-Promoting Ability of Endophytic, Halotolerant Bacteria Associated with *Tetragonia tetragonioides* (Pall.) Kuntze

**DOI:** 10.3390/plants11010049

**Published:** 2021-12-24

**Authors:** Dilfuza Egamberdieva, Jakhongir Alimov, Vyacheslav Shurigin, Burak Alaylar, Stephan Wirth, Sonoko Dorothea Bellingrath-Kimura

**Affiliations:** 1 Leibniz Centre for Agricultural Landscape Research (ZALF), 15374 Muncheberg, Germany; swirth@zalf.de (S.W.); belks@zalf.de (S.D.B.-K.); 2 Faculty of Biology, National University of Uzbekistan, Tashkent 100174, Uzbekistan; jahongir.alimov@gmail.com (J.A.); slaventus87@inbox.ru (V.S.); 3 Department of Molecular Biology and Genetics, Faculty of Arts and Sciences, Agri Ibrahim Cecen University, Agri 04100, Turkey; balaylar@agri.edu.tr; 4 Faculty of Life Science, Humboldt University of Berlin, 10115 Berlin, Germany

**Keywords:** New Zealand spinach, plant-associated bacteria, microbial inoculants

## Abstract

The diversity of salt-tolerant cultivable endophytic bacteria associated with the halophyte New Zealand spinach (*Tetragonia tetragonioides* (Pall.) Kuntze) was studied, and their plant beneficial properties were evaluated. The bacteria isolated from leaves and roots belonged to *Agrobacterium*, *Stenotrophomonas*, *Bacillus*, *Brevibacterium*, *Pseudomonas*, *Streptomyces*, *Pseudarthrobacter*, *Raoultella*, *Curtobacterium*, and *Pantoea*. Isolates exhibited plant growth-promoting traits, including the production of a phytohormone (indole 3-acetic-acid), cell wall degrading enzymes, and hydrogen cyanide production. Furthermore, antifungal activity against the plant pathogenic fungi *Fusarium solani*, *F. oxysporum*, and *Verticillium dahliae* was detected. Ten out of twenty bacterial isolates were able to synthesize ACC deaminase, which plays a vital role in decreasing ethylene levels in plants. Regardless of the origin of isolated bacteria, root or leaf tissue, they stimulated plant root and shoot growth under 200 mM NaCl conditions. Our study suggests that halophytes such as New Zealand spinach are a promising source for isolating halotolerant plant-beneficial bacteria, which can be considered as potentially efficient biofertilizers in the bioremediation of salt-affected soils.

## 1. Introduction

Soil salinity is one of the most severe abiotic factors that degrade agricultural land with substantial detrimental effects on plant growth and yield losses worldwide. According to current reports, approximately 1–2% of fertile lands are degraded to infertile land every year caused by salinization [1,2]. Remediation of saline land is not an easy task, and it often needs a novel approach that does not harm the environment and supports environmental sustainability [3,4]. It is known that halophytes hold potential for the bioremediation of salt-affected land and for restoring or improving soil productivity [5,6]. Halophytes withstand salt stress by producing compatible solutes and regulating stress-responsive genes, modulating reactive oxygen species, and drawing benefits from their associated microbes [7,8]. The plant microbiome has been extensively studied to improve plant growth and stress tolerance under various abiotic stress conditions [9,10,11]. The plant rhizosphere is a complex, dynamic, and nutrient-rich ecosystem for microbes, stimulating colonization and nutrient turnover [12]. There are numerous reports on the diversity of halotolerant bacterial species of genera such as *Acetobacter*, *Azospirillum*, *Arthrobacter*, *Bacillus*, *Pseudomonas*, *Pantoea*, *Rhizobium*, *Serratia*, *Streptomyces*, and *Steretrophomonas* associated with halophytes such as *Salicornia bigelovii* [13], *Halocnemum strobilaceum* [14], *Seidlitzia rosmarinus* [15], *Salicornia brachiate* [16], or *Haloxylon ammodendron* [17].

*Tetragonia tetragonioides* (Pall.) Kuntze is a halophyte known as New Zealand spinach [18], extensively distributed along the coasts of Australia, New Zealand, and Tasmania but also in Argentina and Asian countries [19]. The halophyte *T. tetragonioides* is well adapted to an extreme environment and is also known to have the ability to remove salts from saline land [20]. New Zealand spinach is also consumed as a vegetable or in salads and used for medicinal purposes [21]. Bekmirzaev et al. [22] observed a salt tolerance of *T. tetragonioides* up to 200 mM NaCl and a high potential for removing sodium ions from the soil. Although several studies on growth, salt tolerance, and physiological traits of *Tetragonia tetragonioides* were already published, knowledge about endophytes or rhizobacteria associated with New Zealand spinach and their role in plant stress tolerance is still missing.

Halophyte-associated microbes, either rhizospheric or endophytes, help plants withstand salt stress through the modulation of plant metabolites, synthesis of phytohormones and enzymes such as aminocyclopropane-1-carboxylate (ACC) deaminase, which reduces ethylene synthesis [9,15]. In another study, plant growth-promoting rhizobacterial (PGPR) strains *Acinetobacter* sp. and *Pseudomonas putida* increased salt stress tolerance, plant growth, and antioxidant activities in *Sulla carnosa* [23]. A similar observation was reported by Szymańska et al. [24], where *Pseudomonas stutzeri* associated with halophyte *Salicornia europaea* reduced salt stress in *Brassica napus* L. Accordingly, stress tolerance mediated by endophytic bacteria has been reported for *Acacia gerrardii* [7], *Arthrocnemum macrostachyum* [25] and *Lycium ruthenicum* [26] under harsh environmental conditions. These stress-tolerant microbes have a great biotechnological potential to improve soil productivity and plant health of saline soils under arid conditions.

Here, we hypothesize that the halophyte *Tetragonia tetragonioides* is a source of endophytic, halotolerant bacteria that likely possess plant growth-promoting ability and increase plant tolerance to salt stress. We investigated the diversity of cultivable endophytic bacteria of *Tetragonia tetragonioides* grown under salt stress by using 16S rRNA gene analysis and evaluated microbial traits linked to plant fitness under stress. More profound knowledge of the diversity of halophyte-associated bacteria and understanding mutualistic interactions between hosts and microbes are essential for developing effective microbial inoculants applicable to saline agriculture.

## 2. Results

### 2.1. Endophytic Bacteria Associated with Plant Leaves and Roots

A total of thirty-four bacterial isolates were collected from three samples of New Zealand spinach tissues (*Tetragonia tetragonioides* (Pall.) Kuntze). After eliminating siblings, only ten isolates of endophytic bacteria were left from roots and ten isolates from leaves. The strains were identified using 16S rRNA gene analysis and compared with the closest relatives from GenBank (NCBI). The results are shown in Table 1 for roots and Table 2 for leaves.

**Figure 1:**
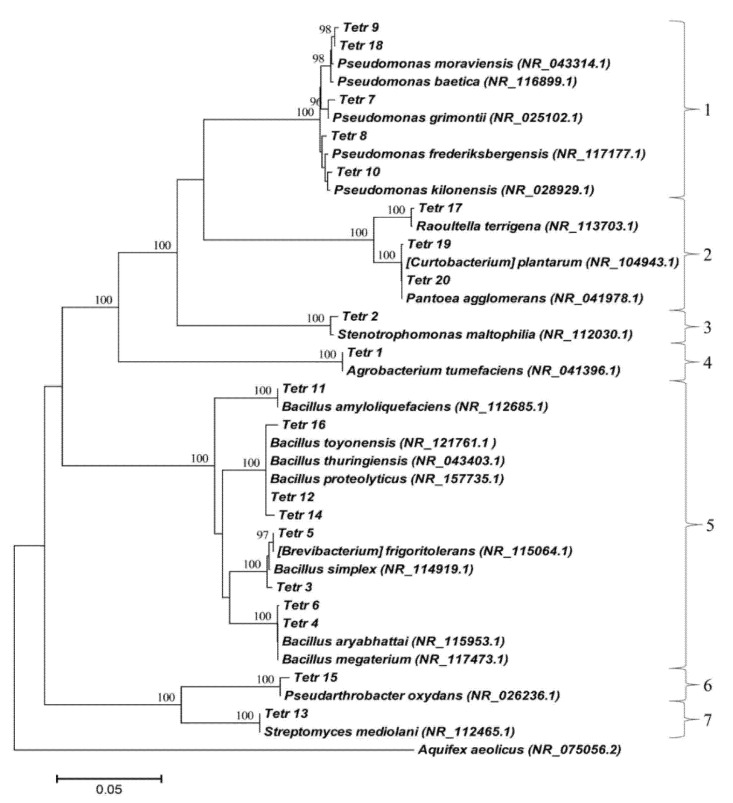
Phylogenetic tree of bacteria endophytes isolated from *Tetragonia tetragonioides* (Pall.) O. Kuntze and their closest relatives from GenBank. The isolates are divided into seven clusters representing orders: 1—Pseudomonadales, 2—Enterobacterales, 3—Xanthomonadales, 4—Hyphomicrobiales, 5—Bacillales, 6—Micrococcales, 7—Streptomycetales.

The length of nucleotide sequences varied from 1390 to 1463 bp for root endophytes and from 1426 to 1465 bp for leaf endophytes. The percent of the identity of the isolated strain to the closest strains from GenBank was between 99.04 and 99.93. The closest relatives of the isolated endophytes are shown in a phylogenetic tree constructed using the Neighbor-Joining method (Figure 1).

The isolated strains belonged to the phyla Firmicutes, Actinobacteria, and Proteobacteria. The most abundant were Proteobacteria with ten bacterial endophytes and Firmicutes with eight species. Only two strains belonged to Actinobacteria. The strains were divided into four classes, i.e., Gammaproteobacteria (9), Bacilli (8), Actinobacteria (2), and Alphaproteobacteria (1). The class of Gammaproteobacteria included the orders Pseudomonadales (Tetr 7, Tetr 8, Tetr 9, Tetr 10, Tetr 18), Enterobacterales (Tetr 20, Tetr 19, Tetr 17), and Xanthomonadales (Tetr 2). The class Bacilli included representatives of three genera, i.e., Bacillus (Tetr 5, Tetr 11, Tetr 12, Tetr 14, Tetr 16), Priestia (Tetr 4, Tetr 6), and Peribacillus (Tetr 3). The class Actinobacteria contained representatives from Streptomycetales (Tetr 13) and Micrococcales (Tetr 15). The class Alphaproteobacteria included just one representative from the order Hyphomicrobiales, the family Rhizobiaceae (Tetr 1).

### 2.2. Plant-Beneficial Traits of Endophytes

The plant-beneficial traits of endophytic bacteria are summarized in Table 3. The bacterial isolates were tested for the production of phytohormone indole-3 acetic acid (IAA), hydrogen cyanide (HCN), as well as for cell wall degrading enzymes (lipase, protease, β-1, 3-glucanase) and ACC deaminase (Table 3).

Seven out of twenty isolates produced HCN, and ten isolates produced ACC deaminase, while the other isolates were negative for ACC deaminase activity. Six isolates produced lipase, eleven isolates protease, and nine isolates β-1, 3-glucanase. Notably, isolate *B. proteolyticus* Tetr 11 produced all three enzymes. Ten isolates produced IAA; the concentration ranged between 2.1 and 5.2 µg mL^−1^. The highest IAA production was observed in isolate *P. moraviensis* Tetr 18 (5.2 ± 0.2 µg mL^−1^), followed by *B. amyloliquefaciens* Tetr 10 (4.8 ± 0.2 µg mL^−1^), and *C. plantarum* Tetr 19 (4.5 ± 0.3 µg mL^−1^).

The antifungal activity of bacterial suspensions was tested against *Fusarium solani*, *F. oxysporum*, and *V. dahliae* (Table 3, Figure S1). Six bacterial isolates showed antifungal activity against the two fungal pathogens. The bacterial isolates *S. maltophilia* Tetr 2, *B. frigoritolerans* Tetr 5, *S. mediolani* Tetr 13, and *P. moraviensis* Tetr 18 inhibited the growth of both *Fusarium* pathogens. *P. grimontii* Tetr 7, *B. amyloliquefaciens* Tetr 11, and *C. plantarum* Tetr 19 were effective against *V. dahliae*. Eleven isolates did not have any antifungal activity.

### 2.3. The Effect of Endophytic Bacteria on Plant Growth

**Figure 2:**
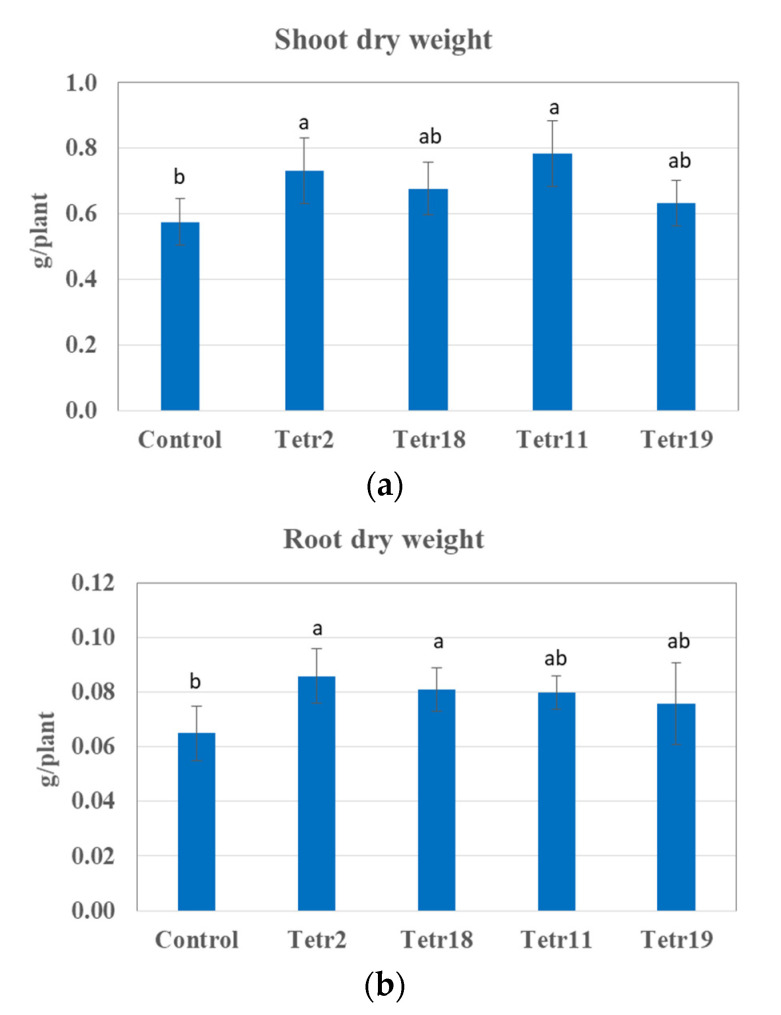
The effect of endophytic bacteria *Stenotrophomonas maltophilia* Tetr 2, *Pseudomonas moraviensis* Tetr 18, *Bacillus amyloliquefaciens* Tetr 11, and *Curtobacterium plantarum* Tetr 19 on shoot (**a**) and root dry weight (**b**) of *Tetragonia tetragonioides* (Pall.) Kuntze. The plants were grown under 200 mM NaCl conditions in a greenhouse for 30 days at 24 °C/16 °C (day/night). Column means with different letters are significantly different based on Tukey’s HSD test at *p* < 0.05.

**Figure 3:**
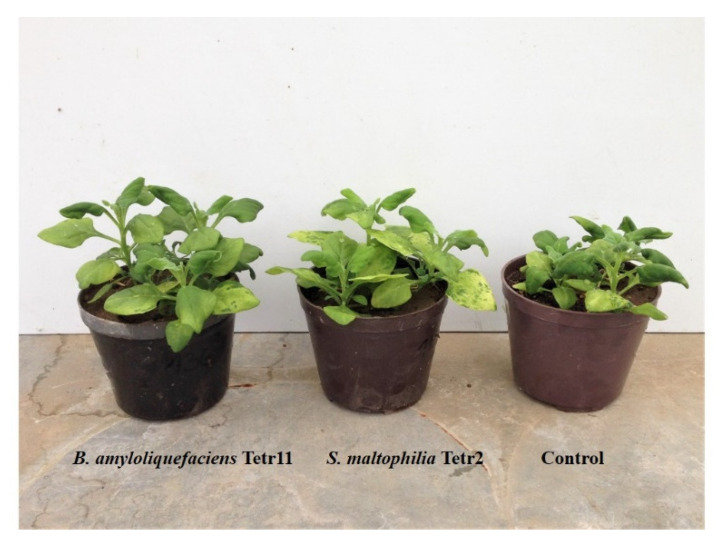
The effect of endophytic bacteria *Bacillus amyloliquefaciens* Tetr 11, and *Stenotrophomonas maltophilia* Tetr 2 on the growth of *Tetragonia tetragonioides* (Pall.) Kuntze. The plants were grown in a greenhouse for 30 days at 24 °C/16 °C (day/night).

The bacterial isolates *S. maltophilia* Tetr 2, *B. amyloliquefaciens* Tetr 11, *P. moraviensis* Tetr 18, and *C. plantarum* Tetr 19 exhibited pronounced plant beneficial traits and were selected for testing their effect on plant growth under saline conditions (200 mM NaCl Figure 2 and Figure 3). The inoculation of seeds with *S. maltophilia* Tetr 2 and *B. amyloliquefaciens* Tetr 11 significantly increased the shoot dry weight by 27 and 35% compared to uninoculated plants exposed to salt stress, respectively (Figure 2a). There was no significant effect after 30 days of plant growth when seeds were inoculated with *P. moraviensis* Tetr 18 or *C. plantarum* Tetr 19. Three bacterial isolates, i.e., *S. maltophilia* Tetr 2, *B. amyloliquefaciens* Tetr 11, and *P. moraviensis* Tetr 18 stimulated root growth of New Zealand spinach from 21 up to 38% as compared to uninoculated plants (Figure 2b). The significant differences were found for shoot growth of plants inoculated with *S. maltophilia* Tetr 2, and *B. amyloliquefaciens* Tetr 11, and for root growth of plants inoculated with *S. maltophilia* Tetr 2, *P. moraviensis* Tetr 18.

### 2.4. Survival of Bacterial Isolates in the Root Tissue

In order to assess whether the bacterial isolates colonize plant root tissue after inoculation, we selected spontaneous rifampicin-resistant mutants from each strain and used them for the re-inoculation test. The results showed that three bacterial isolates *S. maltophilia* Tetr 2, *B. amyloliquefaciens* Tetr 11, and *P. moraviensis* Tetr 18 colonized internal plant root and leaf tissues (Table 4).

## 3. Discussion

Overall, our study is the first report about cultivable endophytic bacteria derived from leaves and roots of New Zealand spinach and their plant-beneficial interactions, to the best of our knowledge. Numerous reports have already been published on the diversity of salt-tolerant plant beneficial bacteria, including various species belonging to *Acetobacter*, *Azospirillum*, *Arthrobacter*, *Bacillus*, *Pseudomonas*, *Rhizobium*, *Serratia*, and *Steretrophomonas* [27,28]. They preferentially colonize the root system rich in nutrients due to exudates and form beneficial associations with the plants [10,22,29]. These microbes adopted various drought and salt stress strategies through several physiological acclimation mechanisms [30].

In our study, we identified *Agrobacterium tumefaciens* Tetr 1 associated with the leaves of New Zealand spinach. *Agrobacterium tumefaciens* was also observed in peach, which exhibits several plant-beneficial traits such as nitrogen fixation, phosphate solubilization, and production of IAA [31]. Accordingly, another salt-tolerant plant, *Sesbania cannabina,* is associated with the genera *Agrobacterium*, *Ensifer*, and *Rhizobium* [32]. Another isolate from our study, *S. maltophilia*, was already observed in saline soil and showed plant growth stimulation of *Arachis hypogaea* under salt stress [33]. In general, most bacterial strains adapted to harsh conditions belong to *Bacillus*. Accordingly, we observed several *Bacillus* species in our study, such as *B. simplex*, *B. aryabhattai*, *B. megaterium*, *B. amyloliquefaciens*, *B. proteolyticus*, and *B. thuringiensis* associated with New Zealand spinach. Kearl et al. [1] reported that species of the genera *Bacillus*, *Halomonas*, and *Kushneria* were also associated with other halophytes such as *Salicornia rubra* A. Nelson, and *Sarcocornia utahensis* (Tidestrom) A.J. Scott. Moreover, several other bacterial species of the genera *Brevibacterium*, *Streptomyces*, and *Pseudomonas* were observed with New Zealand spinach. Furthermore, Shurigin et al. [15] found *Brevibacterium* and *Pseudomonas* associated with the salt-tolerant plant *Seidlitzia rosmarinus* Ehrenb. ex Boiss.

The bacterial isolates exhibited several plant-beneficial traits, including effects on plant growth, physiology, and stress tolerance either directly or indirectly. Half of the studied isolates synthesized the phytohormone IAA as well as ACC deaminase. Our results showed that root-associated bacteria included a higher number of IAA-producing isolates compared with leaf-associated bacteria. Numerous reports on phytohormone production by endophytic bacteria confirm their critical role in maintaining plant health under stress conditions [10,34,35]. Thus, the inoculation of *Sulla carnosa* (Desf.) provenances with *Pseudomonas* sp. improved the root system and shoot growth through IAA production under saline soil conditions [36]. In another study, ABA-producing *Bacillus amylo-liquefaciens* stimulated rice plant growth under saline conditions [37]. Moreover, under stress conditions, the plant produces more ethylene, which negatively impacts root/shoot proliferation and plant development. ACC deaminase produced by endophytic bacteria reduces the ethylene level and helps the plant restore its development. It is well reported that endophytes can relieve plant stress by blocking the pathway of ethylene synthesis in plants [38,39]. Moreover, plant-associated beneficial microbes control soil-borne plant pathogens through several mechanisms, including the production of cell wall degrading enzymes, such as β-1,3-glucanase and lipase, and also protease [9,35,40,41,42,43]. In our study, three bacterial isolates from New Zealand spinach showed antifungal activity against *V. dahliae*, which indicates they may protect the plant from Verticillium wilt caused by *Verticillium dahliae* Kleb. on New Zealand Spinach [44].

In addition, three bacterial isolates *S. maltophilia* Tetr 2, *P. moraviensis* Tetr 18, and *B. amyloliquefaciens* Tetr 11 stimulated plant root and shoot growth under salt stress. There are numerous reports and abundant evidence about improved growth, development, and stress tolerance of plants in natural environments or under induced salinity provided by halotolerant, plant-beneficial endophytic bacteria. Such bacteria, associated with *Psoralea corylifolia* [45] and *Lycium ruthenicum* [46], enhanced stress tolerance and the development of wheat. Halotolerant *B. alcalophilus* and *B. thuringiensis* isolated from *Arthrocnemum macrostachyum* (Moric.) K. Koch improved plant growth through the production of IAA [25]. In another study, *B. endophyticus* and *Arthrobacter egilis* associated with *Saliconia europaea* L. and exhibited ACC deaminase activity, increased plant growth and salt stress tolerance of maize [47]. The biomass and development of another cereal, *Hordeum secalinum*, was increased under salt stress conditions by inoculation of *Curtobacterium flaccumfaciens* [48].

Generally, endophytes are known to colonize plant tissues better than other free-living bacteria that actively colonize plants [49]. All three bacterial isolates that stimulated plant growth could be detected in leaves and root tissues after re-inoculation of plants. Accordingly, the occurrence of similar bacteria in roots and leaves has previously been reported for *Boechera stricta* (Graham) Al-Shehbaz by Wagner et al. [50]. There is evidence that chemotaxis is a process that is critical for bacterial colonization and plays an important role in the migration of bacteria from root to aerial parts of the plant [51].

## 4. Materials and Methods

### 4.1. Plant and Microorganisms

The seeds of *Tetragonia tetragonioides* (Pall.) Kuntze were obtained from University Lille, France. Pots (d = 16 cm) were filled with 1 kg of sandy-loamy soil, derived from the Experimental station of Leibniz Centre for Agricultural Landscape Research (ZALF), Müncheberg, Germany. The soil consists of clay and fine silt (7%), coarse and medium silt (19%), and sand (74%) and is characterized by the following properties: pH 6.2; organic C content 0.55%, total N content 0.07%, P content 0.03%, K content 1.25%, and Mg content 0.18%. The seeds were surface-sterilized with 70% ethanol and 10% v/v NaOCl for 5 min, followed by rinsing with sterile water. The seeds were germinated on paper tissue in a dark room at 25 °C for 5–6 days. Plants were grown in growth chambers at ZALF, Müncheberg, Germany, for 30 days at a temperature of 24 °C/16 °C (day/night) and humidity of 50–60% and irrigated with tap water supplemented with 200 mM NaCl.

### 4.2. Isolation of Endophytic Bacteria

Three individual plants were collected as a whole, and the roots were separated from the stems and rinsed in water to remove the soil attached to the roots. Approximately 10 g of the roots and leaves of each plant were sterilized with 10% NaClO and 70% ethanol. The roots and leaves of three sterile samples were crushed separately with a sterile mortar and then mixed with phosphate buffer solution [52]. Tryptic Soy Agar (TSA) (BD, Difco Laboratories, Detroit, MI, USA) with the addition of nystatin 50 µg mL^−1^ and supplemented with 3% NaCl was used as a nutrient medium for isolation of bacteria from the mixtures of roots and leaves in sterile phosphate-buffered saline. Then, 100 µL of the mixtures from final dilutions (10^−1^–10^−5^) were spread on TSA, and plates were left in a thermostat for 96 h at 28 °C. The plates were checked for bacterial growth, and each colony with a different color, shape, surface, or consistency was considered a source of new isolates and transferred to nutrient agar plates. The roots and leaves were tested for sterility by placing them onto TSA plates.

### 4.3. Identification of Bacteria

The DNA was isolated using the method of heat treatment as described by Dashti et al. [53]. The horizontal gel electrophoresis was applied to test the presence of DNA and its amount and quality using NanoDrop™ One. The polymerase chain reaction (PCR) was used for amplification of the extracted 16S rRNA genes by means of the following primers: 27F 5′-GAGTTTGATCCTGGCTCAG-3′ (Sigma-Aldrich, St. Louis, MO, USA) and 1492R 5′-GAAAGGAGGTGATCCAGCC-3′ (Sigma-Aldrich, St. Louis, MO, USA) [54]. The 16S rRNA products were analyzed for restriction fragment length polymorphism, and siblings among bacterial isolates were reduced as described by Jinneman et al. [55]. The USB^®®^ ExoSAP-IT^®®^ PCR Product Cleanup Kit (Affymetrix, USB^®®^ Products, Santa Clara, CA, USA) was used for the purification of PCR products. The purified PCR products were sequenced using ABI PRISM BigDye 3.1 Terminator Cycle Sequencing Ready Reaction Kit (Applied Biosystems, Foster City, CA, USA). The nucleotide sequences were evaluated, corrected, and aligned using Chromas (v. 2.6.5) software and EMBOSS Explorer, http://emboss.bioinformatics.nl/emboss-explorer/ (accessed on 22 December 2021). The Basic Local Alignment Search Tool was applied to identify the 16S rRNA gene sequences and compared with the closest relatives registered in GenBank of NCBI, http://www.ncbi.nlm.nih.gov/ (accessed on 22 December 2021). The ClustalX 2.1 and MEGA6 software [56] were used to construct the phylogenetic tree (Figure 1). The evolutionary history was inferred using the Neighbor-Joining method [57]. The sum of branch length of the optimal tree was 1.00766267. The percentage of replicate trees in which the associated taxa clustered together in the bootstrap test (500 replicates) are shown above the branches. The evolutionary distances were computed using the Neighbor-Joining method [58] and are in the units of the number of base substitutions per site. The analysis involved 41 nucleotide sequences. All positions containing gaps and missing data were eliminated. There were a total of 1271 positions in the final dataset. The 16S rRNA sequences were deposited in GenBank (NCBI) under the accession numbers MT825572–MT825581 for root endophytes and MT825582–MT825591 for leave endophytes.

### 4.4. In Vitro Screening for Plant Beneficial Traits

The antifungal properties of bacterial isolates were tested against *Fusarium solani*, *Fusarium oxysporum*, and *Verticillium dahlia* (Figure S1). Cell suspensions of bacterial isolates were pre-incubated in TSB medium for 72 h and subsequently poured into pre-cut agar wells placed around a potato dextrose agar disk with fungal inoculum precultivated for four days. The plates were incubated at 27 °C for up to 4–5 days, and the zones of inhibition around the wells were estimated.

The HCN production by bacterial isolates was tested on TSA medium. The color change of filter paper saturated with 1% picric acid and 2% sodium carbonate solutions placed in Petri plates was determined [59]. Protease production was detected by the cultivation of strains on TSA/20 (1/20 part of trypsin soybean broth with 1.5% agar) with the addition of skim milk to a final concentration of 5%. The presence of a halo around colonies indicated protease activity [60]. The method of Walsh et al. [61] was used to determine β-1, 3-glucanase production with lichenan as substrate. A Tween lipase indicator assay was used to determine the lipase activity of bacterial isolates [62]. The production of IAA (indole 3-acetic acid) by endophytic isolates was studied using the method of Bano and Musarrat [63], evaluating IAA production by the detection of pink color after 30 min.

The ability of bacteria to utilize 1-aminocyclopropane-carboxylate (ACC) as the sole N-source was determined by incubating strains in BM minimal broth [64] supplemented with 1.5% NaCl. For testing the use of ACC as a sole N source, the medium was also supplemented with 3.0 mM ACC (Sigma Chemical Co., St. Louis, MO, USA). The medium containing (NH_4_)_2_SO_4_ as a sole N source was used as a positive control, while the medium without an added N-source was a negative control.

### 4.5. The Effect of Bacteria on Plant Growth

The effect of selected bacterial isolates that produced IAA, ACC-deaminase, and had antibiotic activity against pathogenic fungi on the growth of New Zealand spinach was conducted in a growth chamber. The seeds were surface-sterilized as described above and germinated on paper tissue in a dark room at 25 °C for 5–6 days. Selected bacterial isolates, which showed the best plant beneficial traits, were used to inoculate seedlings. The bacterial isolates were grown in Tryptic Soy Broth (BD, Difco Laboratories, Detroit, MI, USA) for two days, and cells were washed with 1 mL sterile phosphate-buffered saline (PBS) (20 mM sodium phosphate and 150 mM NaCl, pH 7.4). Cell suspensions were adjusted to a cell density of 10^8^ cells mL^−1^. Sterilized and germinated seeds were coated with bacteria by soaking them for 10 min. Three seedlings were sown to each pot, and after one week, the seedlings were thinned to two plants per pot. Finally, plants were grown in the greenhouse at a temperature of 24 °C/16 °C (day/night) and irrigated with tap water supplemented with 200 mM NaCl. The treatments included: (i) control plants (uninoculated) and (ii) inoculated with endophytic bacteria. Each treatment consisted of four pots and was arranged in a randomized complete block design. After 30 days, root and shoot dry biomass per individual plant (g) were determined.

### 4.6. Survival of Bacterial Isolates in the Plant Root

The ability of bacterial isolates to endophytically colonize the internal plant tissues of New Zealand spinach were also investigated. The spontaneous mutants of the parental strain *S. maltophilia* Tetr 2, *B. amyloliquefaciens* Tetr 11, and *P. moraviensis* Tetr 18 resistant to rifampicin were obtained by plating onto TSA medium amended with an increasing concentration of rifampicin (5–150 µg mL^−1^). After incubation for five days at 28 °C, antibiotic-resistant colonies having similar colony morphology and growth rate with the parental strain were selected. The plant seeds were sterilized, germinated, and coated with rifampicin-resistant mutants as described previously. Control plants did not receive any bacterial inoculation. Plants were grown in pots as described above for three weeks. At harvest, four plants were selected from each treatment, and the root part was washed. Next, 1 g aliquots from each root and leaf sample were sterilized and crushed with a sterile mortar and mixed with PBS as described above. Then, 100 µL of the mixtures from final dilutions (10^−1^–10^−5^) were spread on TSA supplemented with 150 µg mL^−1^ rifampicin. After incubation for three days at 28 °C, the number of rifampicin-resistant colonies was counted.

### 4.7. Data Analyses

The data were subjected to one-way analysis of variance (ANOVA) using the statistical software package SPSS 22 (SPSS Inc., Chicago, IL, USA). The comparisons between treatments were tested at the *p* < 0.05 level using Tukey’s honestly significant difference (HSD) test. The mean values of IAA production, antifungal activity, and the standard deviations were extracted for each observation.

## 5. Conclusions

To the best of our knowledge, this is the first report of endophytic, halotolerant bacteria derived from the leaves and roots of *Tetragonia tetragonioides* (Pall.) Kuntze. The isolates belong to *Agrobacterium*, *Stenotrophomonas*, *Bacillus*, *Brevibacterium*, *Pseudomonas*, *Streptomyces*, *Pseudarthrobacter*, *Raoultella*, *Curtobacterium*, and *Pantoea*. The isolates exhibited plant growth-promoting traits, including the production of IAA, HCN, and showed antifungal activity against plant pathogenic fungi. Half of the isolated bacteria could synthesize ACC deaminase that plays an essential role in sinking ethylene levels in plant tissues. This study suggests that halophytes such as *Tetragonia tetragonioides* (Pall.) Kuntze are a valuable source for isolating halotolerant plant-beneficial bacteria, which can be considered as potentially efficient biofertilizers in the bioremediation of salt-affected soils.

## Figures and Tables

**Table 1:** Sequence similarities of endophytic bacteria isolated from the root system of *Tetragonia tetragonioides* (Pall.) Kuntze with sequences registered in GenBank.

Isolated Strains Deposited to GenBank			Closest Match (16S Ribosomal RNA Genes) (GenBank)		
Strain	Length (bp)	Accession Number	Species	Accession Number	Percent Similarity
Tetr 1	1391	MT825572	*Agrobacterium tumefaciens*	NR_041396.1	99.71
Tetr 2	1444	MT825573	*Stenotrophomonas maltophilia*	NR_112030.1	99.65
Tetr 3	1390	MT825574	*Bacillus simplex*	NR_114919.1	99.42
Tetr 4	1463	MT825575	*Bacillus aryabhattai*	NR_115953.1	99.66
Tetr 5	1457	MT825576	*[Brevibacterium] frigoritolerans*	NR_115064.1	99.73
Tetr 6	1463	MT825577	*Bacillus megaterium*	NR_117473.1	99.11
Tetr 7	1456	MT825578	*Pseudomonas grimontii*	NR_025102.1	99.45
Tetr 8	1445	MT825579	*Pseudomonas frederiksbergensis*	NR_117177.1	99.24
Tetr 9	1438	MT825580	*Pseudomonas baetica*	NR_116899.1	99.24
Tetr 10	1445	MT825581	*Pseudomonas kilonensis*	NR_028929.1	99.38

**Table 2:** Sequence similarities of endophytic bacteria isolated from leaves of *Tetragonia tetragonioides* (Pall.) Kuntze with sequences registered in GenBank.

Isolated Strains Deposited to GenBank			Closest Match (16S Ribosomal RNA Genes) (GenBank)		
Strain	Length (bp)	Accession Number	Species	Accession Number	ACC
Tetr 11	1450	MT825582	*Bacillus amyloliquefaciens*	NR_112685.1	99.72
Tetr 12	1459	MT825583	*Bacillus proteolyticus*	NR_157735.1	99.79
Tetr 13	1438	MT825584	*Streptomyces mediolani*	NR_112465.1	99.44
Tetr 14	1462	MT825585	*Bacillus thuringiensis*	NR_043403.1	99.45
Tetr 15	1426	MT825586	*Pseudarthrobacter oxydans*	NR_026236.1	99.58
Tetr 16	1465	MT825587	*Bacillus toyonensis*	NR_121761.1	99.45
Tetr 17	1450	MT825588	*Raoultella terrigena*	NR_113703.1	99.10
Tetr 18	1451	MT825589	*Pseudomonas moraviensis*	NR_043314.1	99.04
Tetr 19	1439	MT825590	*[Curtobacterium] plantarum*	NR_104943.1	99.24
Tetr 20	1439	MT825591	*Pantoea agglomerans*	NR_041978.1	99.93

**Table 3:** Traits possibly involved in biocontrol and/or plant growth promotion by bacterial endophytes from *Tetragonia tetragonioides* (Pall.) O. Kuntze.

Strain	Antifungal Activity against Phytopathogenic Fungi (mm) ^1^			Hydrogen Cyanide ^2^	ACC Deaminase ^2^	Production of Exoenzymes ^2^			Indole 3 Acetic-Acid (µg mL^−1^)
	*Fusarium solani*	*Fusarium oxysporum*	*Verticillium dahliae*			Lipase	Protease	β-1, 3-Glucanase	
*Agrobacterium tumefaciens* Tetr 1	no	no	no	−	+	−	+	+	4.8 ± 0.2
*Stenotrophomonas maltophilia* Tetr 2	7 ± 0.1	9 ± 0.2	no	−	+	−	+	+	4.2 ± 0.2
*Bacillus simplex* Tetr 3	no	no	no	+	−	−	−	−	3.9 ± 0.3
*Bacillus aryabhattai* Tetr 4	no	no	no	−	−	−	−	−	2.1 ± 0.3
*Bacillus frigoritolerans* Tetr 5	4 ± 0.2	6 ± 0.1	no	+	+	+	−	−	−
*Bacillus megaterium* Tetr 6	no	no	no	−	−	−	+	+	−
*Pseudomonas grimontii* Tetr 7	no	4 ± 0.1	6 ± 0.1	+	+	−	+	+	4.2 ± 0.2
*Pseudomonas frederiksbergensis* Tetr 8	no	no	no	−	−	−	−	−	3.5 ± 0.2
*Pseudomonas baetica* Tetr 9	no	no	no	+	−	+	+	−	−
*Pseudomonas kilonensis* Tetr 10	8 ± 0.1	no	no	−	−	−	−	−	−
*Bacillus amyloliquefaciens* Tetr 11	no	5 ± 0.1	6 ± 0.1	+	+	−	+	−	4.8 ± 0.2
*Bacillus proteolyticus* Tetr 12	no	no	no	−	+	+	+	+	−
*Streptomyces mediolani* Tetr 13	10 ± 0.1	5 ± 0.1	no	−	+	−	+	−	−
*Bacillus thuringiensis* Tetr 14	no	no	no	−	−	+	−	+	−
*Pseudarthrobacter oxydans* Tetr 15	no	no	no	−	+	+	−	−	3.2 ± 0.3
*Bacillus toyonensis* Tetr 16	4 ± 0.1	no	no		−	−	−	+	−
*Raoultella terrigena* Tetr 17	no	no	no	+	−	−	+	+	−
*Pseudomonas moraviensis* Tetr 18	6 ± 0.1	6 ± 0.1	no	+	+	−	+	+	5.2 ± 0.2
*Curtobacterium plantarum* Tetr 19	no	no	4 ± 0.1	−	+	−	+	−	4.5 ± 0.3
*Pantoea agglomerans* Tetr 20	no	no	no	−	−	+	−	−	−

^1^ The antifungal activity of the bacterial isolates against pathogenic fungi is expressed by the size of the inhibition zone formed, in mm (average ± standard deviation, *n* = 3); “no” indicates that no inhibition zone was formed. ^2^ The symbols (+) and (−) for enzymes and hydrogen cyanide (HCN) signify that they were detected or non-detectable, respectively.

**Table 4:** The colonization of bacterial isolates in root and leaf tissues of *Tetragonia tetragonioides* 21 days after inoculation.

Bacterial Isolates	Log CFU g^−1^ Plant Tissue	
	Root	Leaf
*S. maltophilia* Tetr 2	3.92 ± 0.17	3.51 ± 0.17
*B. amyloliquefaciens* Tetr 11	4.26 ± 0.12	3.72 ± 0.19
*P. moraviensis* Tetr 18	4.03 ± 0.20	3.59 ± 0.23

Mean ± standard deviation of four replicates; CFU—colony-forming units.

## Data Availability

Not applicable.

## Supplementary Materials

The following are available online at https://www.mdpi.com/article/10.3390/plants11010049/s1, Figure S1: Antifungal activity of bacterial isolates against Fusarium oxysporum (A) and bacterial colonies on agar plates (B) (*S. maltophilia* Tetr 2 (a); *B. amyloliquefaciens* Tetr 11 (b); *P. moraviensis* Tetr 18 (c)).
