# Supplementary material for: Diversity and Plant Growth-Promoting Ability of Endophytic, Halotolerant Bacteria Associated with Tetragonia tetragonioides (Pall.) Kuntze
Source: Plants (Basel). 2021 Dec 24;11(1):49. doi: 10.3390/plants11010049 (PMC8747539; doi:10.3390/plants11010049)
Supplement: Supplementary file 1 [file plants-11-00049-s001.zip › plants-1427459-supplementary.pdf]

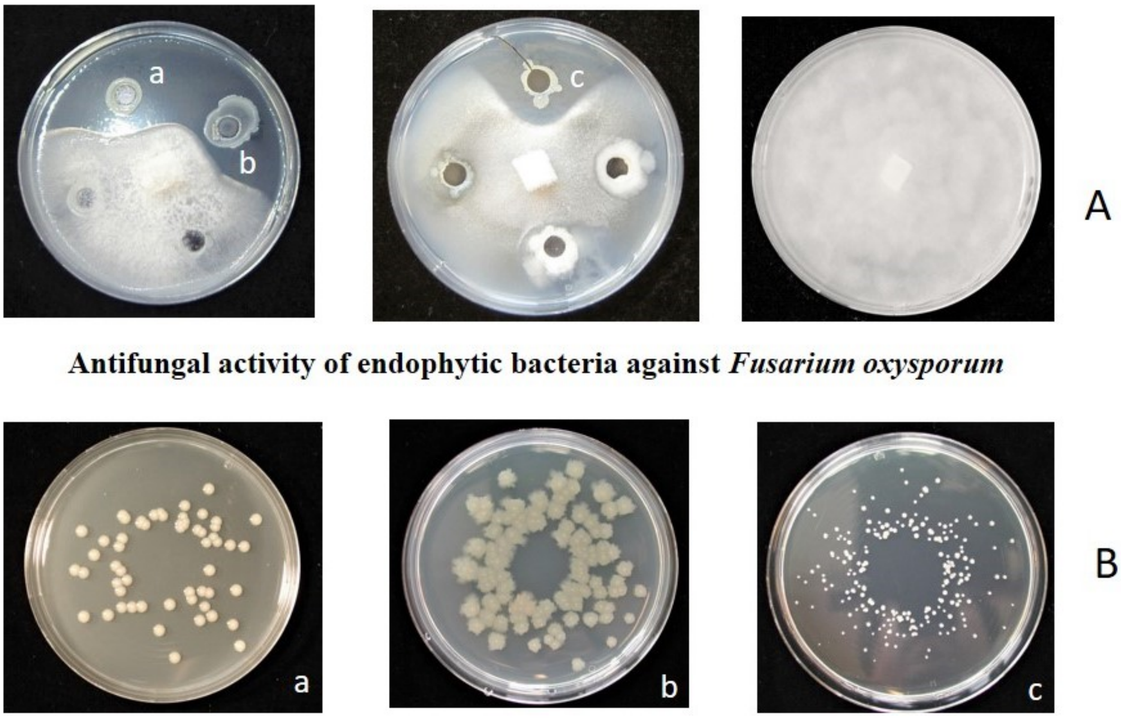

Antifungal activity of endophytic bacteria against *Fusarium oxysporum*

Bacterial colonies on agar plates

***S. maltophilia* Tetr 2 (a)** **_B. amyloliquefaciens_ Tetr 11 (b)** **_P. moraviensis_ Tetr 18 (c)**

Figure S1. Antifungal activity of bacterial isolates against *Fusarium oxysporum* (A) and bacterial colonies on agar plates (B) (*S. maltophilia* Tetr 2 (a); *B. amyloliquefaciens* Tetr 11 (b); *P. moraviensis* Tetr 18 (c))
